# RIPK3 interacts with MAVS to regulate type I IFN-mediated immunity to Influenza A virus infection

**DOI:** 10.1371/journal.ppat.1006326

**Published:** 2017-04-14

**Authors:** Jeffrey Downey, Erwan Pernet, François Coulombe, Benoit Allard, Isabelle Meunier, Joanna Jaworska, Salman Qureshi, Donald C. Vinh, James G. Martin, Philippe Joubert, Maziar Divangahi

**Affiliations:** 1 Department of Medicine, Department of Pathology, Department of Microbiology & Immunology, McGill University Health Centre, McGill International TB Centre, Meakins-Christie Laboratories, McGill University, Montreal, Quebec, Canada; 2 Department of Pathology, Quebec Heart and Lung Institute, Quebec, Quebec, Canada; University of Southern California, UNITED STATES

## Abstract

The type I interferon pathway plays a critical role in both host defense and tolerance against viral infection and thus requires refined regulatory mechanisms. RIPK3-mediated necroptosis has been shown to be involved in anti-viral immunity. However, the exact role of RIPK3 in immunity to Influenza A Virus (IAV) is poorly understood. In line with others, we, herein, show that *Ripk3*^*-/-*^ mice are highly susceptible to IAV infection, exhibiting elevated pulmonary viral load and heightened morbidity and mortality. Unexpectedly, this susceptibility was linked to an inability of RIKP3-deficient macrophages (Mφ) to produce type I IFN in the lungs of infected mice. In Mφ infected with IAV *in vitro*, we found that RIPK3 regulates type I IFN both transcriptionally, by interacting with MAVS and limiting RIPK1 interaction with MAVS, and post-transcriptionally, by activating protein kinase R (PKR)—a critical regulator of IFN-β mRNA stability. Collectively, our findings indicate a novel role for RIPK3 in regulating Mφ-mediated type I IFN anti-viral immunity, independent of its conventional role in necroptosis.

## Author summary

Influenza A virus (IAV) is a pulmonary pathogen that presents a significant threat to human health through seasonal epidemics and occasional, highly lethal pandemics. Type I IFN is an essential component of anti-viral immunity to influenza infection and pulmonary macrophages are the major source of type I IFN. Recently, we have shown that programmed macrophage cell death (apoptosis) plays a key role in immunity to influenza infection. Interestingly, another cell death program, termed necroptosis, has been also implicated in control of viral infection via receptor-interacting serine/threonine protein kinase 3 (RIPK3). In the present study, we define a novel role of RIPK3 in regulating the type I IFN pathway to protect against lethal IAV infection, which is independent of necroptosis. RIPK3 regulates type I IFN signaling at both the transcriptional and post-transcriptional levels to sequester viral replication and protect the host against influenza infection. Thus, manipulation of the RIPK3 pathways poses a novel avenue in the development of anti-IAV treatments.

## Introduction

Pulmonary macrophages (Mφ) reside in the unique extraepithelial environment of the lower airways and are the main source of one of the key components of host anti-viral immunity: type I IFN (primarily IFN-α and β) [1–3]. However, many highly pathogenic viruses, including IAV, have evolved to reach the lower respiratory tract and effectively sidestep the type I IFN pathway in Mφ. Initial recognition of IAV-ssRNA occurs by the cytosolic RNA helicase retinoic acid-inducible gene I (RIG-I) that interacts with the mitochondrial anti-viral-signaling protein (MAVS) to activate the interferon regulatory factor 3 (IRF3)-mediated type I IFN pathway, upstream of the TANK-binding kinase 1 (TBK1) [4]. Subsequent binding of type I IFNs to their heterodimeric receptor (IFNαR) leads to activation of the JAK/STAT pathway and the transcription of IFN-inducible genes (ISGs), such as the double-stranded RNA-dependent protein kinase R (PKR), which is critical in controlling viral replication, by regulating proteins involved in inhibiting both host and viral translation [5] as well as IFN-β mRNA integrity [6].

Resident alveolar Mφ are the first immune cells to encounter IAV in the airways and orchestrate the immune response [7]. While both the frequency and number of resident alveolar Mφ are constant shortly after infection [8], the frequency and total cell number of bone marrow derived-monocytes, recruited in a CCR2-dependent manner, are significantly increased and represent the major source of Mφ in the lungs during IAV infection [9, 10]. We, and others, have shown that the induction of type I IFN by pulmonary Mφ is indispensable during IAV infection [1, 2, 10]. Thus, it is not surprising that IAV has evolved several strategies to inhibit the type I IFN axis, including encoding the virulence factor PB1-F2, which specifically targets mitochondria to induce early apoptosis in Mφ [11, 12] to limit the production of type I IFN [13]. Interestingly, the receptor interacting serine/threonine protein kinase (RIPK) family members (RIPK1 and RIPK3) regulate necroptosis (a form of programmed necrosis) and play a critical role in immunity to viral infections. For example, RIPK3-mediated necroptosis was shown to be important in the host defense against vaccinia virus [14], murine cytomegalovirus (MCMV) [15], as well as IAV [16]. Additionally, a cell death-independent role for RIPK1 and RIPK3 in inflammation has also been described in myeloid cells. In models of LPS-induced inflammation using bone marrow-derived Mφ (BMD-Mφ) [17], or bone marrow-derived dendritic cells (DC) [18], RIPK3-deficient cells failed to release pro-inflammatory cytokines [19, 20]. Consistent with these studies, it has been also demonstrated that RIPK1 regulates the production of potent inflammatory cytokines, including TNF-α [21]. Importantly, it has recently been shown that RIPK3 confers enhanced viral clearance and protection to IAV by modulating apoptotic and necroptotic cell death in infected lung structural cells [16], while its expression moderately affects the pro-inflammatory and anti-viral signature of fibroblasts [22]. However, the function of RIPK3 in lung immune cells, which contribute significantly to immunity to IAV infection has not been well understood.

In this report, we sought to further delineate the role of RIPK3 in immunity to pulmonary IAV infection. Herein, we define RIPK3 as an essential component of host defense against IAV infection. Surprisingly, RIPK3-deficient mice were extremely susceptible to IAV infection due to a significant reduction in type I IFN. Pulmonary Mφ from RIPK3-deficent mice failed to mount an effective type I IFN response to IAV. Mechanistically, we demonstrated that RIPK3 was upregulated in IAV-infected Mφ and its induction was required for optimal production of type I IFN at two steps: via interaction with MAVS to regulate IFN-β transcription and via activation of PKR to stabilize IFN-β mRNA. Notably, the loss-of-function in RIPK3 has no effect on cell death responses to IAV-infected Mφ, both *in vitro* and *in vivo*, indicating a new cell-death independent function for RIPK3 in innate anti-viral responses.

## Results

### RIPK3-deficient mice are highly susceptible to IAV infection and exhibit a heightened pulmonary viral load

**Fig 1:**
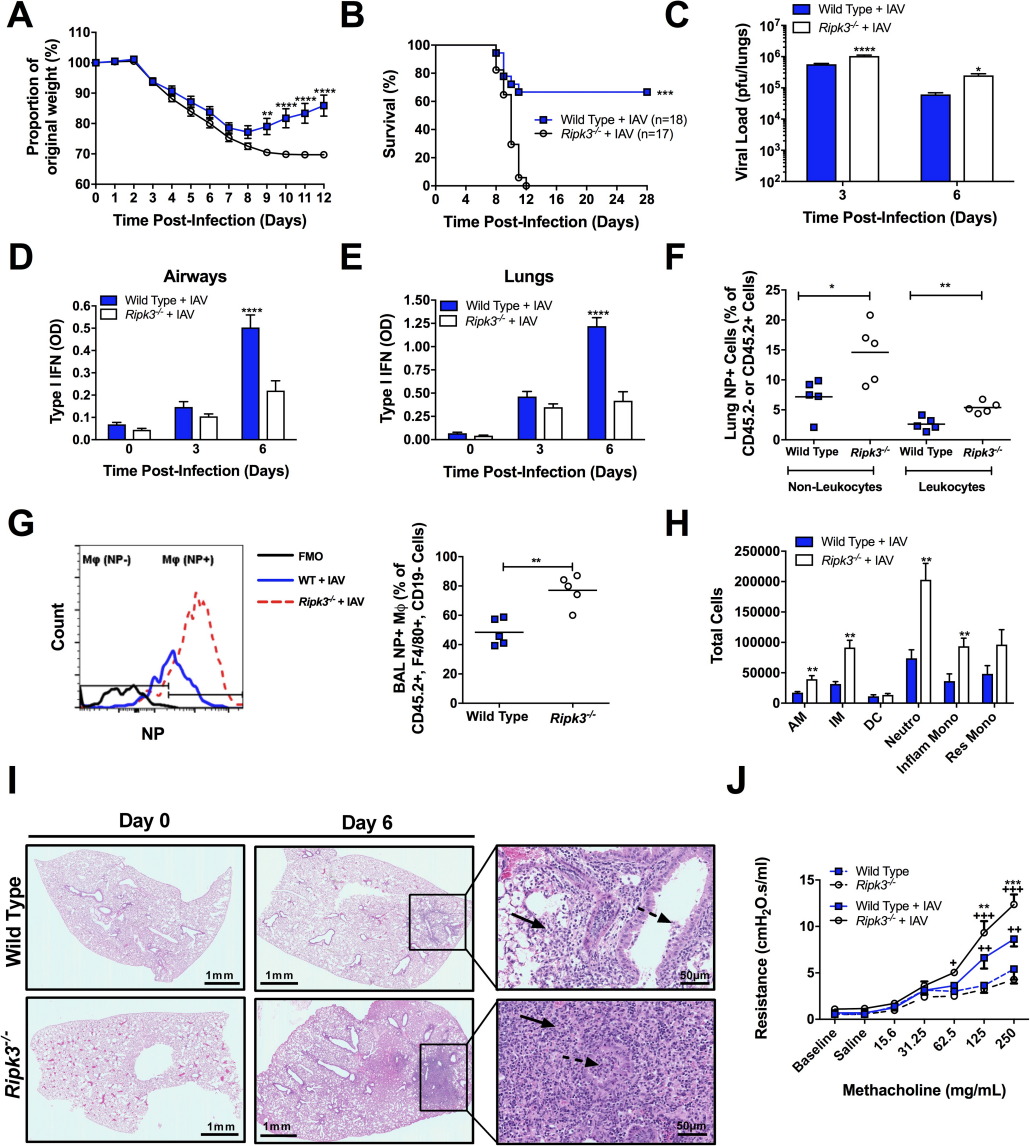
RIPK3 restricts early viral replication and prevents excessive inflammation, morbidity, and mortality during IAV infection. (A-J) WT and *Ripk3*^*-/-*^ mice were infected with a sublethal dose (50 pfu) of IAV and morbidity, as a percentage of original weight (A), and survival (B) were assessed. Pulmonary viral loads (C) and total active type I IFN (α and β) via B16-blue reporter cells in the bronchoalveolar lavage (BAL) (D) or lung parenchyma (E) were measured at various times post-infection. (F-G) At 3 days post-infection lungs and BAL from WT and *Ripk3*^*-/-*^ mice were collected and cells were intracellularly stained for IAV NP protein. (F) Percentage of NP^+^ non-leukocytes and leukocytes in the lung. (G) Representative histogram (left panel) of NP protein levels in Mφ (CD45.2^+^ F4/80^+^ CD19^-^ cells) of the BAL and the frequency of NP^+^ Mφ (right panel). (H) Number of alveolar Mφ (AM), interstitial Mφ (IM), dendritic cells (DC), neutrophils (Neutro), Gr1^+^ inflammatory monocytes (Inflam Mono), and Gr1^-^ resident monocytes (Res Mono) present in the BAL at day 3 post-infection. (I) Micrographs of H&E-stained lung sections prepared prior to and 6 days after IAV infection. At low power, inflammation is absent in both Wild Type and *Ripk3*^*-/-*^ (day 0). At high power, the inflammatory infiltrate is composed of lymphocytes, histiocytes and neutrophils within the alveolar space (solid arrow) and bronchiolar lumen (dotted arrow), shown at 6 days post-infection. Scale bar represents 1mm (low magnification) and 50μm (higher magnification). Using flexivent, total respiratory resistance (J) of uninfected or IAV-infected mice was measured following methacholine challenge at day 6 post-infection. Data are represented as mean ± SEM. *p<0.05, **p<0.01, ***p<0.001, ****p<0.0001 between genotypes as indicated, in J, † indicate significant differences over baseline parameter readings of the same genotype. Except in A and B (as indicated), n = 4–8 animals per group per time point.

To examine a potential role for RIPK3 in immunity to IAV infection, wild-type (WT) and *Ripk3*^-/-^ mice were infected with a low dose (50 pfu) of IAV. *Ripk3*^-/-^ mice exhibited significant morbidity as shown by increased weight loss (Fig 1A) as well as mortality (Fig 1B) compared to WT mice. Similar data were obtained using a higher dose of (90 pfu ≈LD_50_) of IAV infection (S1A and S1B Fig). This increase of mortality was associated with a significantly increased pulmonary viral load (Fig 1C) and decreased levels of active type I IFN in both the airways (Fig 1D) and the lungs (Fig 1E and S1C Fig). Corresponding to the increased pulmonary viral load (Fig 1C), *Ripk3*^*-/-*^ lungs had a higher frequency of viral nucleoprotein (NP)^+^ cells in both epithelial cells (Non-leukocytes, CD45^-^ NP^+^) as well as leukocytes (CD45^+^ NP^+^) (Fig 1F). Interestingly, the percentage of NP^+^ pulmonary Mφ (CD45^+^ F4/80^+^ CD19^-^) was higher in the lung (S1D Fig) and BAL (Fig 1G) of *Ripk3*^*-/-*^ mice, indicating RIPK3-deficient Mφ are more susceptible to IAV infection *in vivo*. The initial control of virus propagation is a major determinant of an adequate host immune response, allowing elimination of the pathogen with minimal immunopathology. In line with this, the significant increase in viral load in *Ripk3*^*-/-*^ mice correlated with markedly enhanced inflammation (Fig 1H, S2A and S2B Fig) and immunopathology (Fig 1I and S1E Fig) as well as reduced pulmonary function (Fig 1J). Collectively, these data indicate that RIPK3 plays an indispensable role in immunity to IAV infection by regulating host pulmonary anti-viral responses and reducing pulmonary immunopathology.

### RIPK3 is required for optimal induction of type I IFN in Mφ infected with IAV

**Fig 2:**
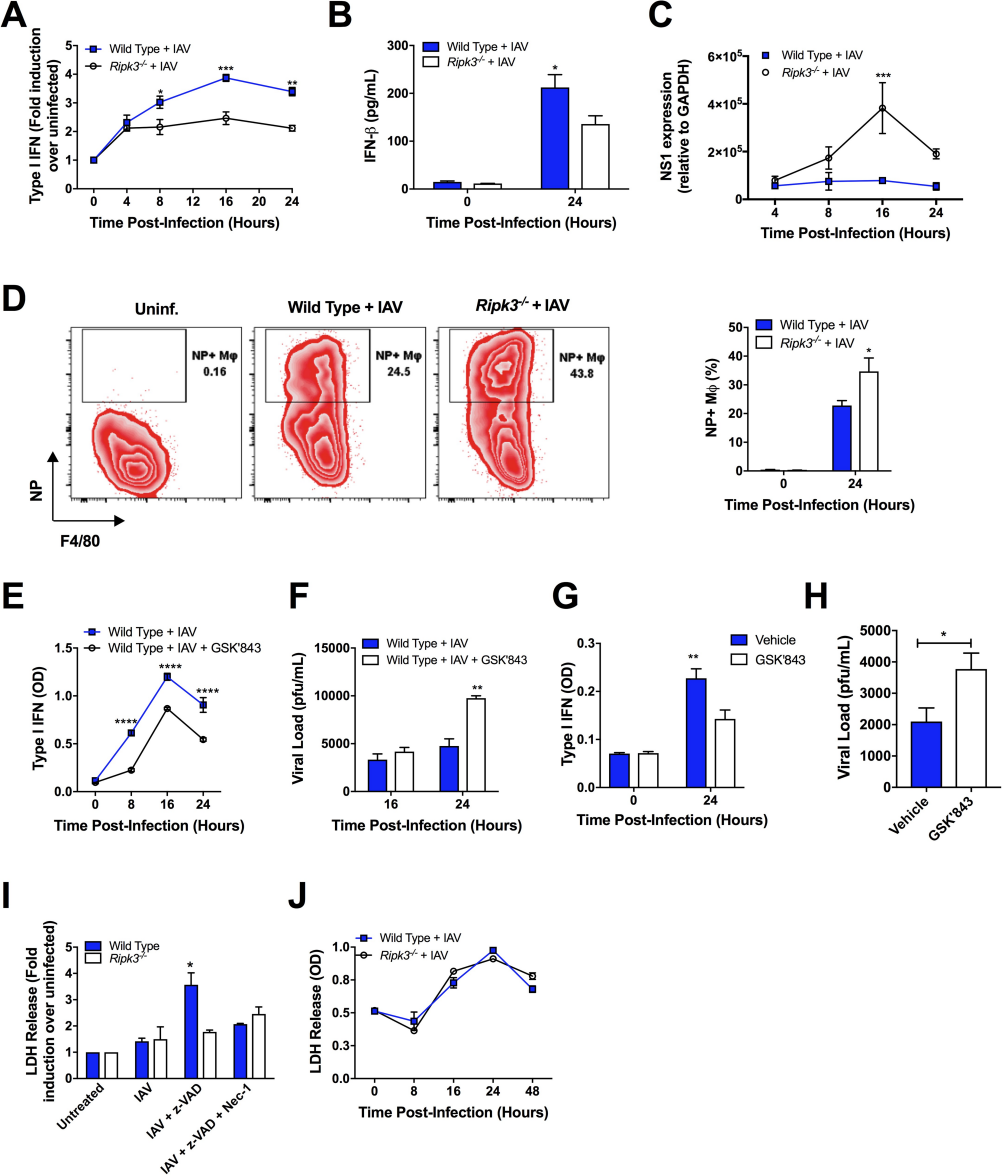
RIPK3-deficient BMD-Mφ are impaired in anti-viral immunity, independent of the necroptosis pathway. (A-F, I-J) BMD-Mφ from WT and *Ripk3*^*-/-*^ mice were generated and infected with IAV at MOI 1. Total active type I IFN (α and β) (A) and IFN- β (B) was assessed in the supernatants. (C) The relative levels of viral NS1 mRNA were determined via qPCR. (D) BMD-Mφ from WT and *Ripk3*^*-/-*^ mice were infected with IAV and the level of viral protein NP was analyzed by flow cytometry. Zebra plots (left panel) are representative of the 24h time-point and numbers adjacent to the gates indicate percent of NP^+^ Mφ as quantified in the right panel. Level of total active type I IFN in cell culture supernatants (E) and viral load (F) in BMD-Mφ from WT mice treated, or not, with the selective RIPK3 inhibitor GSK‘843 (10μM) and infected with IAV. (G-H) Human monocyte-derived Mφ treated, or not, with the selective RIPK3 inhibitor GSK‘843 (10μM) were infected with IAV H3N2. Levels of active type I IFN (G) and viral load (H) were assessed in culture supernatants 24h after infection. (I) BMD-Mφ from WT and *Ripk3*^*-/-*^ mice were generated and treated with various combinations of zVAD-FMK (zVAD, 25μM) and necrostatin-1 (Nec-1, 10μM) for 1h and then were infected with IAV. Necroptosis was assessed by lactate dehydrogenase (LDH) assay after 24h of IAV infection. (J) LDH was measured in BMD-Mφ cell culture supernatants following IAV infection at various time points. Data are representative of the mean ± SEM of triplicate wells and are representative of at least 3 experiments. *p<0.05, **p<0.001, ***p<0.001, ****p<0.0001

During the steady state, the pulmonary compartment is primarily comprised of resident alveolar Mφ (AMφ). However, after pulmonary infection the recruitment of monocyte/Mφ from the bone marrow is critical for host defense to infection [23]. Since Mφ are the primary source of type I IFN in response to pulmonary viral infections [1–3, 10] and *Ripk3*^*-/-*^ mice elicited attenuated type I IFN responses to IAV, we next determined whether *Ripk3*^*-/-*^ Mφ are impaired in their ability to produce type I IFN *in vitro*. Consistent with the significant reduction of type I IFN and increased viral load in the lungs of RIPK3-deficient mice, a significant reduction of total active type I IFN and IFN-β was observed in IAV-infected *Ripk3*^*-/-*^ BMD-Mφ (Fig 2A and 2B), AMφ (S3A Fig), but not BMDC (S3B Fig). In addition, RIPK3-deficient BMD-Mφ were more permissive to IAV infection, as evaluated by qPCR for IAV NS1 transcripts (Fig 2C), flow cytometry for NP protein (Fig 2D), or standard plaque assay (S3C Fig). Similarly, WT BMD-Mφ treated with the selective inhibitor of RIPK3 activity (GSK ‘843) also exhibited less active type I IFN upon IAV infection (Fig 2E) with a higher viral load (Fig 2F). Finally, we sought to extend the role of RIPK3 in immunity to IAV in human monocyte-derived Mφ. Using monocyte-derived Mφ generated from peripheral blood mononuclear cells (PBMC) obtained from healthy donors and infected with a human strain of IAV (H3N2), RIPK3-inhibited PBMC released significantly less type I IFN (Fig 2G) and exhibited an elevated viral load (Fig 2H). These data collectively indicate that RIPK3 regulates the induction of type I IFN in murine and human Mφ infected with IAV.

As necroptosis has been shown as a mechanism involved in cytokine release (e.g. IL-1) [19], and RIPK3 is a key player in this cell death pathway, we initially hypothesized that RIPK3-mediated necroptosis is required for the secretion of type I IFN from IAV-infected BMD-Mφ. Consistent with other experimental models using LPS [24], which show that the addition of the pan-caspase inhibitor (zVAD-FMK) is required for the induction of necroptosis (S3D Fig), we also found that inhibition of caspases via zVAD increased necroptosis in IAV-infected BMD-Mφ (Fig 2I). In these experimental models, the induction of necroptosis was RIPK1- and RIPK3-dependent, as necroptosis was completely abrogated by addition of necrostatin-1 (Nec-1) or the loss-of-function of RIPK3 (Fig 2I and S3D Fig). However, in the absence of zVAD, using the LDH assay (Fig 2J), Annexin V/7-AAD staining (S3E Fig), or viability dye (S3F Fig), we found no difference in the cell death program between groups of IAV-infected BMD-Mφ at any time points after infection. In agreement with these *in vitro* observations, the levels of pulmonary Mφ death were not altered in the BAL of WT and *Ripk3*^*-/-*^ mice infected with IAV (S3G Fig). Thus, the use of pan-caspase inhibitors to reveal necroptosis in the majority of experimental models may mask the other biological functions of RIPK1/RIPK3 in natural settings. Together, these results indicate that the lack of type I IFN responses by *Ripk3*^*-/-*^ BMD-Mφ is not coupled to cell death outcomes in IAV-infected Mφ.

### RIPK3 interacts with MAVS, mediating type I IFN signaling in BMD-Mφ infected with IAV

**Fig 3:**
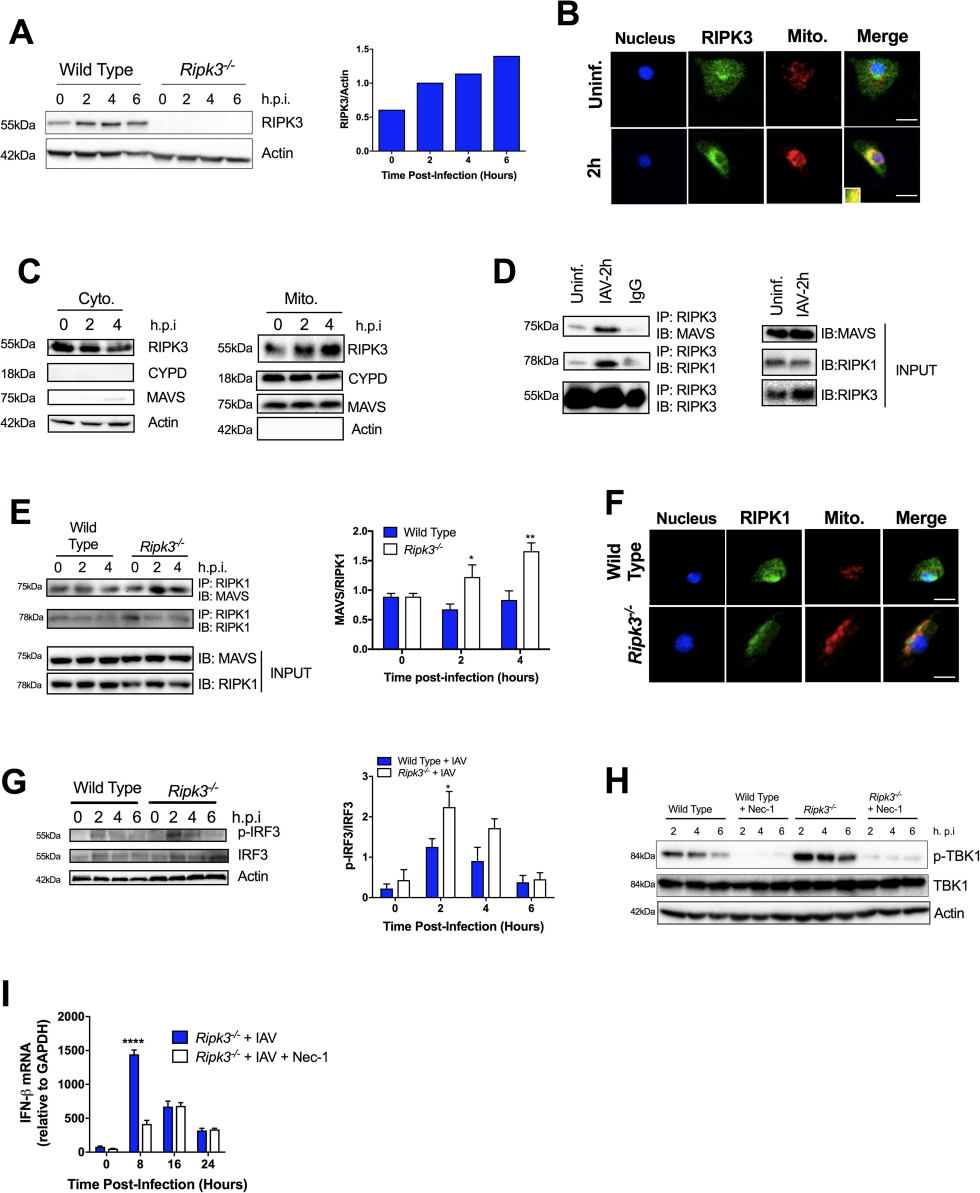
RIPK3 interacts with MAVS in IAV-infected BMD-Mφ regulating TBK1/IRF3 dependent type I IFN pathway. BMD-Mφ were infected with IAV at an MOI of 5 (A-H) or 1 (I). (A) Western blot analysis of RIPK3 expression at various times post-IAV infection in WT BMD-Mφ. Densitometry analysis to quantify ratio of RIPK3 to β-actin is shown in the right panel. (B) Immunofluorescence analysis of co-localization of RIPK3 (green) and mitochondria (red) in WT BMD-Mφ infected or not with IAV. Yellow regions are the areas of RIPK3 and mitochondria colocalization. Nuclei were stained with Hoechst (blue). The scale bars represent 10μm. (C) BMD-Mφ lysates were collected at 0, 2 and 4 hours post-IAV infection. Cytosolic and mitochondrial fractions were isolated and analyzed by western blot for RIPK3 and MAVS. Actin and mitochondrial protein CYPD were used as loading controls and to ensure purity of the fractions. (D-E) BMD-Mφ lysates were collected at 0, 2 (D-E) and 4 (E) hours post-IAV infection and immunoprecipitation was performed with anti-RIPK3 (D) or anti-RIPK1 (E). Samples were then analyzed by immunoblotting for MAVS or RIPK1. (E, right panel) Densitometry analysis to quantify the interaction between MAVS and RIPK1 is shown, representative blot in left panel (n = 3). (F) Immunofluorescence analysis of co-localization of RIPK1 (green) and mitochondria (red) in WT and *Ripk3*^*-/-*^ BMD-Mφ 2 hours post IAV-infection. Nuclei were stained with Hoechst (blue). The scale bars represent 10μm. (G) Representative blot (left panel) of phosphorylated IRF3 in WT and *Ripk3*^*-/-*^ BMD-Mφ infected, or not, with IAV at various times post-infection. Densitometry analysis to quantify ratio of phosphorylated IRF3 to total IRF3 is shown in the right panel (n = 3). (H-I) WT and *Ripk3*^*-/-*^ BMD-Mφ were pretreated with/without necrostatin-1 (Nec-1, 10μM) for 1h and then were infected, or not, with IAV. Representative blot of the phosphorylation of TBK1, determined by western blot as in B. (I) Total RNA was extracted and the expression of IFN-β mRNA was determined by qPCR. Data are expressed as mean ± SEM representative of at least three independent experiments. *p<0.05, **p<0.001, ****p<0.0001.

As the induction of the type I IFN pathway is critically mediated by RIG-I/MAVS signaling during IAV infection in BMD-Mφ (S4A Fig), we next assessed whether RIPK1 and RIPK3 are involved in regulation of this pathway. Following IAV infection of WT BMD-Mφ, we observed an upregulation of RIPK3 (Fig 3A). Additionally, we found that prior to IAV infection, RIPK3 is primarily localized in the cytoplasm (Fig 3B and 3C). However, following IAV infection of WT BMD-Mφ, the levels of cytoplasmic RIPK3 markedly decreased, while there was an increase in RIPK3 translocation to the mitochondria (Fig 3B and 3C). Interestingly, we also found that RIPK3 interacted with MAVS (Fig 3D and S4B Fig) and surprisingly, in IAV-infected *Ripk3*^*-/-*^ BMD-Mφ, there was a robust interaction between RIPK1 and MAVS (Fig 3E and S4B and S4C Fig) at the mitochondria (Fig 3F). Furthermore, this enhanced interaction between RIPK1/MAVS led to a significantly increased level of phosphorylation of the downstream mediators of MAVS signalling, TBK1 and the transcription factor IRF3 (Fig 3G and 3H). A similar trend was also observed in stimulation of *Ripk3*^*-/-*^ Mφ with the RIG-I ligand, 5’triphosphate (ppp) dsRNA, which led to increased levels of interaction between RIPK1 and MAVS, increased phosphorylation of IRF3, as well as IFN-β transcripts (S4D–S4F Fig). To test whether RIPK1 directly mediates TBK1 activation, we next inhibited RIPK1 using Nec-1 and demonstrated that TBK1 activation was completely abolished in IAV-infected *Ripk3*^*-/-*^ BMD-Mφ (Fig 3H and S4G Fig). Moreover, inhibition of RIPK1 by Nec-1 significantly decreased the levels of IFN-β mRNA after IAV infection (Fig 3I). These data collectively indicate that in the absence of RIPK3, there is increased activation of the RIPK1-dependent RIG-I/MAVS signaling pathway. Additionally, these findings support that RIPK3 plays as a negative regulator of RIPK1-mediated type I IFN signaling pathways during IAV infection.

### RIPK3 activates the PKR pathway in IAV-infected BMD-Mφ, increasing the integrity of IFN-β transcripts and promoting protection

**Fig 4:**
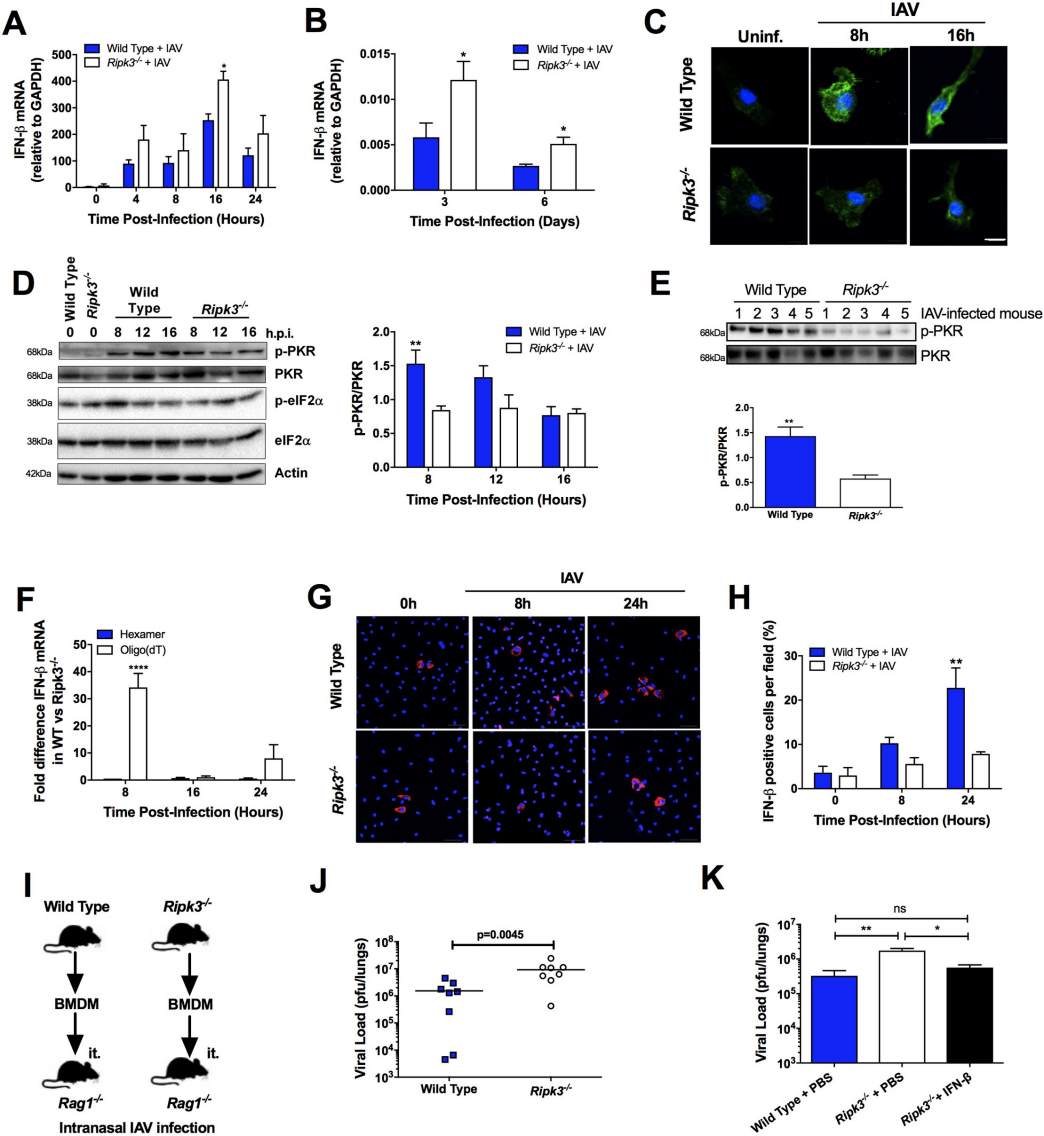
RIPK3 regulates IFN-β mRNA integrity through activation of PKR. Total RNA was extracted from IAV-infected BMD-Mφ (MOI 1) (A) or cells of the BAL (50 pfu) (B) and the expression of IFN-β mRNA was determined by qPCR. (C) Phosphorylation of PKR (green) was analyzed by immunofluorescence in WT and *Ripk3*^*-/-*^ BMD-Mφ at different time point post IAV-infection. Nuclei were stained with Hoechst (blue). The scale bars represent 10μm. (D) Phosphorylated and total forms of PKR and eIF2α in whole-cell lysates were analysed by immunoblotting. β-Actin was used as a loading control. One representative blot is shown (left panel). Densitometry analysis to quantify the ratio of phosphorylated PKR relative to total PKR (n = 4, right panel). (E) Cells were harvested from the BAL of infected (50 pfu) WT or RIPK3-deficient mice and levels of phosphorylated and total PKR were determined by western blot (top panel). Densitometry analysis to quantify the ratio of phosphorylated PKR relative to total PKR is shown in the bottom panel. (F) Difference in the expression of IFN-β mRNA between WT and *Ripk3*^*-/-*^ BMD-Mφ infected with IAV. Gene expression was analyzed by qPCR following cDNA generation using random hexamers (blue bars) or oligo(dT) primers (white bars). (G) Confocal images showing IFN-β production in IAV-infected BMD-Mφ. Cells were stained with a rabbit polyclonal antibody specific for IFN-β (red) as well as nuclear dye Hoechst (blue). (H) Percentage of cells positive for IFN-β per random field. The scale bars represent 50μm. (I) BMD-Mφ (1x10^6^ cells) from WT and *Ripk3*^*-/-*^ mice were adoptively transferred (i.t.) into naïve *Rag1*^*-/-*^ mice, which were then infected with 500 PFU of IAV 2h post-transfer. (J) Viral load was assessed 3 days after IAV-infection (n = 8, compilation of 2 experiments). (K) Wild Type and *Ripk3*^*-/-*^ mice were infected with 50 pfu of IAV. After 2 days, mice were intranasally administered PBS or 2000U of IFN-β. Viral load was determined at day 3 post-infection by standard plaque assay (n = 7–8 mice/group, compilation of 2 experiments). *p<0.05 **p <0.01, ****p <0.0001, ns = not significant.

Given the increased activation of TBK1/IRF3 signaling in *Ripk3*^*-/-*^ BMD-Mφ, we next investigated whether this leads to an upregulation at the transcriptional level of IFN-β. In line with the increased TBK1/IRF3 signaling, IFN-β transcripts were elevated in IAV-infected RIPK3-deficient BMD-Mφ (Fig 4A). Similarly, the levels of IFN-β transcripts were also significantly elevated in the lungs of *Ripk3*^*-/-*^ mice after 3 and 6 days of IAV infection (Fig 4B). These results were surprising as the levels of IFN-β protein were significantly reduced in both IAV-infected *Ripk3*^*-/-*^ BMD-Mφ (Fig 2A and 2B) and lungs (Fig 1D and 1E and S1C Fig). To address the disparity between the transcription and translation of IFN-β in RIPK3-deficient BMD-Mφ and lungs, we next investigated the mechanism involved in IFN-β post-transcriptional regulation. Protein kinase R (PKR) is an important player in the host response to viral infections mainly via phosphorylating the α subunit of the translation initiation factor eIF2 (eIF2α) that inhibits both host and viral mRNA translation, thus suppressing viral propagation [5, 25]. Additionally, several studies also demonstrate that PKR plays a key role in augmenting type I IFN responses to viral infection [6, 26]. The mechanism of PKR function is either at a translational level through activation of eIF2α kinase [27] or at a post-transcriptional level by preserving the integrity of IFN-β mRNA, via the maintenance of its poly(A)-tail [6]. We found that during IAV infection there was no difference in phosphorylation of eIF2α in *Ripk3*^*-/-*^ or WT BMD-Mφ (Fig 4D). However, after IAV infection, activation of PKR was markedly reduced in *Ripk3*^*-/-*^ BMD-Mφ, compared to the WT as assessed by confocal microscopy (Fig 4C) or western blot (Fig 4D), and no significant effects were observed in the total PKR protein (S5A Fig). Correlating to these *in vitro* findings, we also found a significant reduction of PKR phosphorylation in the lungs of IAV-infected *Ripk3*^*-/-*^ mice, compared to infected WT mice (Fig 4E). Furthermore, the reduction of PKR activation in IAV-infected *Ripk3*^*-/-*^ BMD-Mφ correlated with diminished IFN-β mRNA stability, as evaluated by the levels of IFN-β mRNA after synthesis of cDNA with oligo(dT) primers, compared to hexamer primers (Fig 4F). Comparable to another viral model [6], this effect was specific to IFN-β mRNA, as the levels of GAPDH (S5B Fig) and IL-6 (S5C Fig) mRNA did not differ following synthesis with either hexamer or oligo(dT) primers. In agreement with the loss of IFN-β mRNA integrity, confocal analysis confirmed the reduction of intracellular IFN-β protein levels within *Ripk3*^*-/-*^ IAV-infected BMD-Mφ (Fig 4G and 4H). These data collectively indicate that in RIPK3-deficient Mφ and mice infected with IAV, PKR activation is profoundly compromised, leading to reduced IFN-β mRNA stability and thus IFN-β production.

Finally, to directly address whether the reduced anti-viral function of *Ripk3*^*-/-*^ BMD-Mφ *in vitro* translates to an impaired control of IAV replication *in vivo*, we adoptively transferred (intratracheally) BMD-Mφ from either *Ripk3*^*-/-*^ or WT mice into *Rag1*-deficient mice (lacking B and T cells), which were then infected intranasally with IAV (Fig 4I). At day 3 post IAV-infection, the *Rag1*^*-/-*^ mice that received *Ripk3*^*-/-*^ BMD-Mφ showed a significantly increased pulmonary viral titre in comparison to the *Rag1*^*-/-*^ mice that received WT BMD-Mφ (Fig 4J). Finally, to provide the direct link between reduction of type I IFN in RIPK3-deficient mice and susceptibility to IAV infection, we reconstituted IFN-β in the lungs of *Ripk3*^*-/-*^ mice and showed that there was a significant reduction in pulmonary viral load, which was comparable to the viral load in infected WT mice (Fig 4K). Taken together, our data provide the first evidence that RIPK3 intrinsically regulates anti-viral immunity in Mφ, independent of its conventional role in necroptosis, by driving PKR activation and the IFN-β anti-viral effector program.

## Discussion

**Fig 5:**
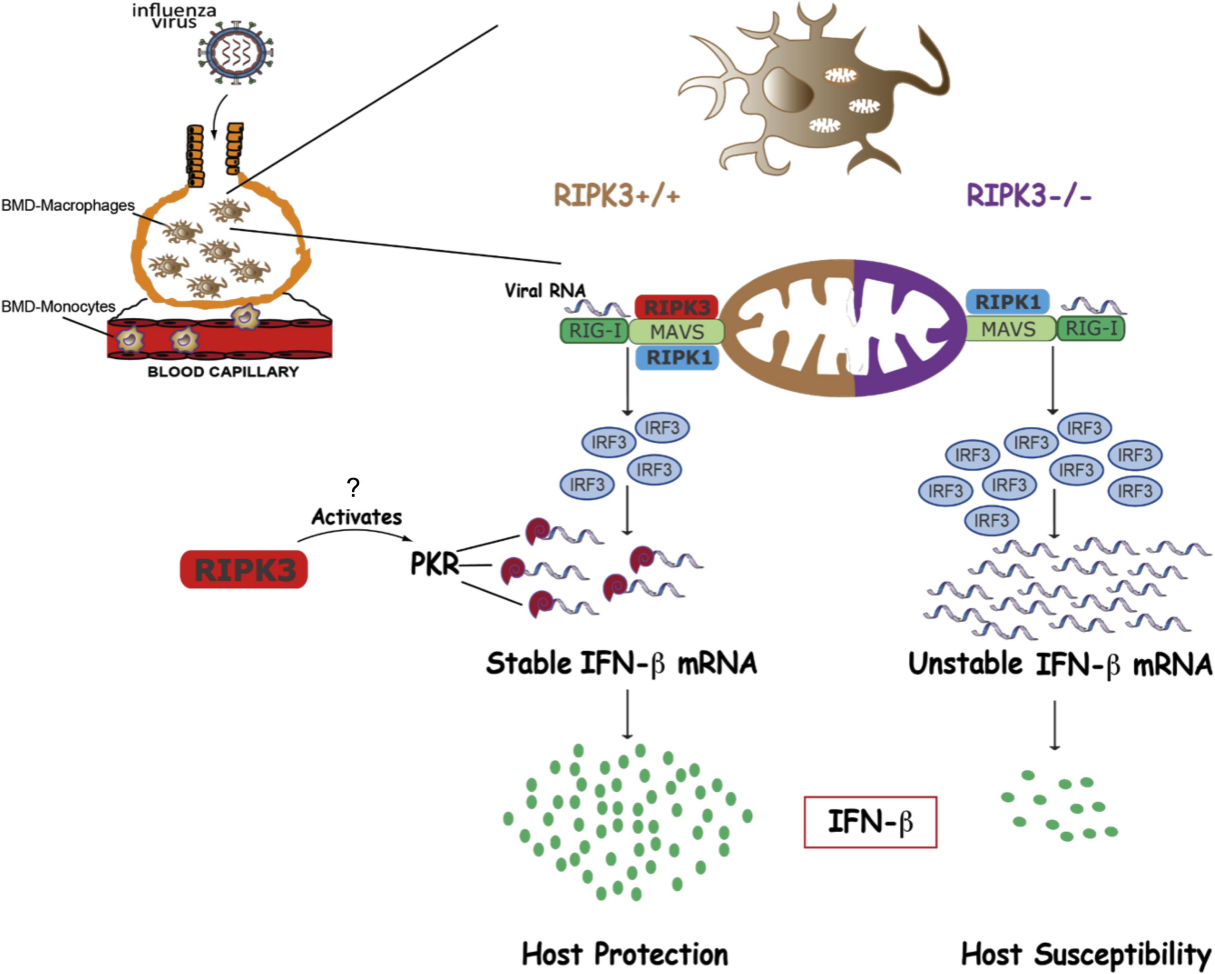
RIPK3 enhances innate anti-viral immunity against Influenza A virus. Pulmonary infection by IAV triggers the recruitment of monocytes from the bone marrow that differentiate into macrophages. IAV encounters and infects those macrophages, where viral RNA activates the RIG-I/MAVS pathway, leading to production of the key anti-viral cytokine IFN-β. IAV-induced RIPK3 interaction with MAVS at the mitochondria and may represent an immune evasion strategy to decrease IFN-β production. In the absence of RIPK3, there is increased RIPK1/MAVS interactions, which enhance downstream signaling, resulting in higher TBK1/IRF3 activation and IFN-β mRNA levels. However, this mechanism is counteracted by the RIPK3-mediated activation of PKR. PKR stabilizes IFN-β mRNA through the poly(A) tail, leading to increased IFN-β protein production and, ultimately, host protection.

In the present study, we define a novel and critical role of RIPK3 in host defense against IAV. Our findings provide strong evidence that in IAV-infected Mφ, RIPK3 regulates type I IFN production at both the transcriptional level, via interaction with the RIG-I/MAVS signaling pathway, as well as the post-transcriptional level, via activation of PKR (Fig 5).

RIPK3 was initially identified as a master regulator of necroptosis [28]. Genetic studies have undoubtedly shown the critical physiological role of RIPK1/3 dependent necroptosis in embryonic development [29, 30]. Moreover, accumulating evidence indicates that RIPK1/3 are also key players in host defense. Several viral infections have been shown to initiate RIPK1/3 mediated necroptosis, which contributes to host immunity against the infection [14, 15, 31]. While a previous study suggested that the protection and survival of RIPK3-deficient mice is comparable to WT mice following IAV infection [32], here we have demonstrated that RIPK3-deficient mice are remarkably susceptible to pulmonary IAV infection, which is also in line with a recent study by Balachandran’s group [16]. The exact nature of this difference is unknown, but we speculate that the strain of IAV, as well as the low dose of IAV (~0.4 LD_50_), which was weight-adjusted, may potentially explain these differences. Additionally, following IAV infection, it was shown that RIPK3 was critical in the production of IL-1β by Mφ via the NLRP3 inflammasome [33]. Although, the *in vivo* consequences of this deficiency were not investigated in that study, activation of the NLRP3 inflammasome was previously shown to be crucial in immunity to IAV infection [34].

RIPK3 deficient mice are fully resistant to murine cytomegalovirus (MCMV) [15], murine hepatitis virus [35] and lymphocytic choriomeningitis virus [36], but they are particularly susceptible to vaccinia virus [14]. The susceptibility of RIPK3-deficient mice to vaccinia has been directly linked to necroptosis in a RIPK1-dependent manner [14], while their resistance to MCMV—despite their inability to induce necroptosis—was RIPK1-independent [15]. These differences might be the reflection of a dual regulatory role of RIPK1 in the transcription of cytokines, as well as cell death. For instance, RIPK1 was shown to be essential in inducing inflammatory cytokines (IL-6, IL-1β, TNF-α) in response to bacterial [37] and viral infections [38]. This pro-inflammatory role may be explained by its ability to trigger NF-κB activation via a TLR3/TRIF-dependent pathway [39, 40]. In the case of type I IFN, during dsRNA responses [38] and following VSV or Sendai virus infection [41], RIPK1 has been shown to be involved in upregulation of type I IFN signaling and can interact with the RIG-I/MDA5/MAVS complex [4, 42, 43]. Similar to these studies, our data indicate that during IAV infection, in the absence of RIPK3, there is a markedly increased interaction between RIPK1 and MAVS compared to WT that leads to enhanced activation of TBK1/IRF3 and transcription of IFN-β mRNA in Mφ. Interestingly, others have previously reported that RIPK3 negatively regulates the TRIF-RIPK1-induced NF-κB pathway [39]. Our data suggest a potentially similar mechanism in which the interaction of RIPK3 with MAVS limits its interaction with RIPK1 to dampen TBK1/IRF3 activation. Whether RIPK3 directly inhibits RIPK1 recruitment to RIG-I/MAVS, or recruitment of other partners in the complex, requires further investigation. Taken together, our data support the function of RIPK1 as an activator of host immunity, while RIPK3 serves to limit RIPK1 activity, regulating the inflammatory response at the signaling level during the “tug-of-war” between host defense and tolerance.

Several recent reports [17, 18, 33, 44] describe the functional role of RIPK3 in regulating pro-inflammatory cytokines, independent of necroptosis. Herein, we showed that RIPK3 controls IFN-β production at the mRNA level, by regulating the stability of IFN-β transcripts through the activation of PKR. It is well established that PKR-deficient cells are impaired in type I IFN production following viral infections. Interestingly, a study by Schulz and colleagues revealed that PKR activation is indispensable in the production of IFN-β to MDA5-mediated viruses (e.g. ECMV) but dispensable for RIG-I-mediated viruses (e.g. Sendai virus, ΔNS1-IAV) in infected DC. However, another study indicated that the optimal production of type I IFN in pulmonary macrophages infected with IAV was dependent on activation of PKR [45]. Similarly, we also found that PKR plays an indispensable role in the stability of IFN-β mRNA by maintaining the poly(A) tail in IAV-infected BMD-Mφ, rather than controlling IFN-β production through eIF2α kinase activation. How RIPK3 regulates PKR activation and which molecular mechanisms are involved upstream of RIPK3 in this process need further investigation.

Interestingly, the DNA-dependent activator of IFN regulatory factors (DAI) is a recently characterized sensor of IAV that modulates both cell death responses [46] and inflammation [47]. In the study by Thapa et al., DAI was shown to promote apoptosis and necroptosis upstream of RIPK3 in IAV-infected fibroblasts, supporting DAI as an activator of RIPK3. Moreover, DAI was first identified as an activator of TBK1/IRF3 in the type I IFN response to herpes simplex virus 1 [48]. These studies are intriguing and may reveal the differential role of RIPK3 in regulating type I IFN production via sensing IAV genomic RNA and the RIG-I/MAVS axis versus necroptosis via DAI/MLKL axis. Potentially, the differential expression of RIG-I versus DAI in different cell types can dictate the functional role of RIPK3 in immune cells (e.g. macrophages) versus structural cells (e.g. fibroblasts or epithelial cells). Furthermore, the replicative capacity of IAV and the levels of cytosolic IAV genomic RNA in each cell type may preferentially activate one axis versus the other. Certainly, this is a very exciting area of research and further investigation is required to determine the mechanisms regulating innate immunity to IAV infection.

Moreover, although in the current study we demonstrate that the function of RIPK3 is dispensable in Mφ death modality during IAV infection, the contribution of RIPK3 in pathogenesis of IAV *in vivo* is certainly more complex and we cannot exclude its potential role as a death kinase in other immune cells or structural cells. In this context, a recent publication highlighted the critical role of RIPK3-mediated necroptosis in promoting immunity to IAV in fibroblasts [16]. Interestingly, they also showed that the levels of type I IFN, although modest, were significantly reduced in RIPK3-deficient fibroblasts after infection with IAV [22]. However, fibroblasts contribute substantially less to type I IFN production than macrophages, which have been demonstrated to be the main producer of type I IFN during IAV infection [10]. Both our study, as well as Balachandran’s studies indicate that RIPK3 has evolved to promote viral clearance through distinct mechanisms in immune (macrophages) and lung structural cells (fibroblasts). Furthermore, the function of RIPK3 also appears to differ among immune cells. For instance, RIPK3-deficient DC were impaired in the production of pro-inflammatory cytokines following LPS stimulation, while Mφ were not [18]. Our data also support this notion since the production of type I IFN was only impaired in Mφ but not DC. Thus, the mechanisms underlying the differential activation of RIPK3 is certainly cell, as well as pathogen specific.

Pulmonary Mφ convert into highly active cells following detection of IAV viral particles by PRRs and become the major source of type I IFN [10]. As the initial control of virus propagation through type I IFN is a major determinant of an adequate host immune response that eliminates the pathogen with minimal immunopathology, IAV has evolved multiple strategies to subdue Mφ type I IFN pathways. We have recently demonstrated that the mitochondrial PRR belonging to the NOD-like family (NLRX1) plays a critical role in Mφ by maintaining mitochondrial fitness and preventing IAV-induced cell death to maximize type I IFN production [13]. IAV’s strategies for suppressing type I IFN in Mφ is not limited only to PRRs, as they also target Mφ eicosanoid pathways [10]. In line with this, one may envision a scenario where IAV evolved a strategy to upregulate RIPK3 to dampen RIPK1/MAVS-mediated TBK1/IRF3/type I IFN production to facilitate its replication. However, the host may have counter-evolved to promote a secondary role of RIPK3 in activating PKR and eliciting type I IFN responses through mRNA stability. Additional studies are required to address this unique function of RIPK3 in regulating type I IFN responses, following infection with other strains of IAV or pathogens.

In summary, we provide *in vivo* evidence showing that the lack of RIPK3 limits the production of type I IFN, which results in enhanced IAV propagation, early excessive host inflammatory responses that contribute to pulmonary tissue and vasculature damage/dysfunction and, ultimately, enhanced mortality. The integrity of type I IFN pathways is essential in anti-viral immunity and identification of molecular mechanisms that are involved in maintaining this will undoubtedly provide new opportunities for targeted therapy of highly pathogenic strains of IAV.

## Materials & methods

### Mice

Six- to ten-week-old C57BL/6 mice were purchased from Jackson Laboratories. *Ripk3*^*-/-*^, kindly provided from Vishva Dixit (Genentech, San Francisco), and *Mavs*^*-/-*^, a kind gift from Dr. Salman Qureshi (McGill University), were bred at McGill University. Experiments were performed using age- and sex-matched mice.

### Isolation and culture of primary macrophages and cell lines

Murine Bone Marrow-Derived Macrophages (BMD-Mφ) were isolated following aseptic flushing of tibiae and femurs of eight- to ten-week-old mice. Macrophages were differentiated from bone marrow precursors for 7 days in RPMI-1640 supplemented with 30% (vol/vol) L929 cell- [American Type Culture Collection (ATCC)] conditioned medium, 10% (vol/vol) FBS, 2 mM L-glutamine, 1 mM sodium pyruvate, 1% essential and nonessential amino acids, 10mM HEPES and 100 U/mL penicillin/streptomycin. To generate BMDC, bone marrow was cultured in RPMI-1640 supplemented with 10% (vol/vol) FBS, 2 mM L-glutamine, 1 mM sodium pyruvate, 1% nonessential amino acids, 100 U/mL penicillin/streptomycin and 0.35% Β-mercaptoethanol, containing 20 ng/ml of GM-CSF as described previously [49]. Alveolar macrophages (AMφ) were collected by bronchoalveolar lavage of naïve mice using cold, sterile PBS. AMφ were cultured in RPMI-1640 supplemented with 10% (vol/vol) FBS, 2 mM L-glutamine, 10mM HEPES and 100 U/mL penicillin/streptomycin. After 1h adhesion, AMφ were washed with PBS and placed in fresh media. Madin-Darby Canine Kidney cells (MDCK) were obtained from ATCC and maintained in Dulbecco’s Modified Eagle Medium (DMEM) enriched with 10% (vol/vol) FBS, 2mM L-glutamine, and 100 U/mL of penicillin/streptomycin. To generate human monocyte-derived macrophages, peripheral blood mononuclear cells (PBMCs) were isolated from healthy donors using Ficoll-Paque PLUS (GE Healthcare, Burlington, ON, Canada), according to the manufacturer’s protocol. PBMCs were then cultured in RPMI with 2% human serum with 20ng/mL of human M-CSF. Monocytes were differentiated for 7 days with fresh media added every second day. All reagents and supplements pertaining to cell culture were purchased from GIBCO. Cells were seeded at a density 0.5–1.5x10^6^ cells/well of a 6-well plate.

### Viruses and infections

All *in vitro* and *in vivo* infections were performed using influenza A/Puerto Rico/8/34 (H1N1) virus (IAV), kindly provided by Dr. Jonathan A. McCullers (St. Jude Children Research Hospital), except for *in vitro* infections of human cells that were performed using the clinical strain H3N2 A/Hong-Kong/1/68. Mice were challenged intranasally (in 25μL PBS) with IAV at a sublethal dose of 50 pfu or a lethal dose of 90 pfu. *In vitro*, BMD-Mφ were seeded in tissue culture plates the day before infection, unless indicated otherwise, and infections were performed in fresh medium lacking L929 cell-conditioned medium with 1, or 5 multiplicities of infection (MOI) of virus. Viruses were propagated and isolated from MDCK cells and titrated using standard plaque assay in MDCK cells [50].

### Flow cytometry

BAL were collected by cannulating the trachea with a 22-gauge cannula, then washing the lungs with 3 x 800μL of cold, sterile PBS. The total volume of the recovered lavage after 3 washes was ~2mL. Cells were initially surface stained with anti-CD16/32 (BD Bioscience) in 0.5% BSA/PBS solution to block non-specific AB interaction with Fc receptors. Cells were then surface-stained with different combinations of PE-conjugated anti-Siglec-F, PE-Cy7-conjugated anti-F4/80, APC-conjugated anti-CD11c, APC-Cy7 anti-CD11b, FITC-conjugated anti-Gr1, PE-Cy5.5-conjugated anti-CD115 (All from BD Biosciences). For NP staining, cells were fixed and permeabilized using BD CytoFix/CytoPerm (BD #554714) before intracellular staining with FITC-conjugated anti-NP (Abcam #ab20343). Flow cytometry was performed using BD LSR II (BD Biosciences) with FACSDiva Software Version 6.1.2 (BD Biosciences). Analysis was performed using FlowJo Software Version 10.0.6 (Tree Star).

### Histopathological analysis

Lungs were inflated and fixed for at least 24 hours with 10% formalin, and then embedded in paraffin. 5 μm sections were cut and stained with hematoxylin-eosin. Slides were scanned at a resolution of 200X magnification (Nanozoomer scanner, Hammamatsu, Japan) and pictures were taken using NDPI viewer (Hammamatsu, Japan).

### Analysis of pulmonary function

Airway responses to methacholine were evaluated using a small animal ventilator (flexiVent apparatus and flexiVent 5.1 software) as previously described [51].

### Cell death analysis

LIVE/DEAD Fixable Violet Dead Cell staining was implemented to determine cell viability following viral infection (Molecular Probes). Necrosis and apoptosis levels *in vitro* and *in vivo* were assessed using the PE-AnnexinV and 7-amino-actinomycin D (7-AAD) Apoptosis Detection Kit I (BD Biosciences) according to the manufacturer’s instructions and analyzed by flow cytometry. Lactate dehydrogenase (LDH) release in culture supernatants of IAV-infected BMD-Mφ was quantified using the CytoTox 96 Non-Radioactive Cytotoxicity Assay (Promega), as per the manufacturer’s recommendations.

### BMD-Mφ stimulation and cytokine quantification

BMD-Mφ were stimulated with 100ng/mL of LPS (Sigma-Aldrich) for 24 hours or with 1μg/mL of 5’triphosphate (ppp) dsRNA (InvivoGen) for different lengths of time. Secretion of total active type I IFN (both IFN-α and IFN-β) in BAL fluid, lung homogenates and cell culture supernatants was assessed using B16-blue IFNα/β reporter cell line for murine samples or HEK-blue IFNα/β reporter for human samples (InvivoGen), according to the specifications of the manufacturer. IFN-β levels in culture supernatants were measured using Verikine Mouse IFN-β ELISA kit (PBL Assay Science #42400–1). When indicated, BMD-Mφ were pre-treated with a combination of necrostatin-1 inhibitor (Nec-1, 10μM, Sigma-Aldrich), zVAD-FMK (zVAD, 25μM, R&D) for 1 hour before infection with IAV. In some experiments, BMD-Mφ were pre-treated with the selective RIPK3 inhibitor GSK’843 (kindly provided by GSK) (10μM) [52]. Samples were then collected for further analysis.

### RNA isolation and RT-qPCR

RNA from BAL of IAV-infected mice or from BMD-Mφ was extracted using Qiazol reagent (Qiagen) according to manufacturer’s instructions. Five hundred ng of RNA was reverse transcribed using the Quantitect Reverse Transcription kit (Qiagen), as directed by the manufacturer. cDNA was generated by qPCR using EvaGreen SYBR Green (Biorad) and the following primers: *GAPDH*-forward: 5’-GGTCCTCAGTGTAGCCCAAG-3’; *GAPDH*-reverse: 5’-AATGTGTCCGTCGTGGATCT-3’; *Ifn-β*-forward: 5’-AGACTATTGTTGTACGTCTCC-3’; *Ifn-*β-reverse: 5’-CAGTAATAGCTCTTCAAGTGG-3’; *IL-6*-forward: 5’-CACAAAGCCAGAGTCCTTCAGAGA-3’; *IL-6*-reverse: 5’-CTAGGTTTGCCGAGTAGATCT-3’-forward. Viral *NS1-*forward: 5’-AGAAAGTGGVAGGCCCTCTTTGTA-3’. Viral *NS1-*reverse: 5’-GGGCACGGTGAGCGTGAACA-3’. Cq values obtained on CFX96 PCR System (Biorad) were analyzed using the formula 2^-ΔCq^ formula normalizing target gene expression to *GAPDH*. In some experiments oligo dT primers (0.4μg/mL, Qiagen) were used in place of random hexamer primers to generate cDNA targeting specifically the poly(A)-tail of the mRNA. For the experiments involved in mRNA stability, the fold difference in gene expression was calculated by using the formula 2^-ΔCq^, where ΔCq = Cq-target gene_Wild-type_−Cq-target gene_*Ripk3*_^-/-^.

### Western blot

Cells obtained from BAL of WT or *Ripk3*^*-/-*^ mice at day 3 post-infection or BMD-Mφ were lysed in lysis buffer (1% Triton X-100, 150mM NaCl, 20mM Hepes pH7.5, 10% glycerol, 1mM EDTA, supplemented with anti-protease and anti-phosphatase cocktails, Roche) and protein concentration was determined using BCA assay (Pierce). 20 μg of protein were resolved by SDS-PAGE and transferred onto PVDF membranes (Biorad). Membranes were blocked and incubated overnight at 4°C with gentle agitation with primary antibodies. The following primary antibodies were used: anti-RIPK3 (1:1000, Proscience #2283), anti-phospho-PKR (1:200, Santa Cruz Biotechnology #sc-101784), anti-PKR (1:200, Santa Cruz Biotechnology #sc-1702), anti-RIPK1 (1:1000, Cell Signaling Technology #3493), anti-phospho-IRF3 (1:500, CST #4947), anti-IRF3 (1:1000, CST #4302), anti-phospho-eIF2α (1:1000, CST #3597), anti-eIF2α (1:1000, CST #5324), anti-MAVS (1:1000, CST #4983), anti-TBK1 (1:1000, CST #3504), anti-pTBK1 (1:1000, CST #13498), anti-CYPD (1:1000, Calbiochem #AP1035) and anti-actin (1:10000, Sigma-Aldrich #2066). Primary antibodies were followed by HRP-conjugated secondary antibodies and signal was detected using Clarity ECL kit (Biorad) and acquired on Chemidoc MP System (Biorad). Densitometry analyses were performed using ImageJ software (NIH).

### Mitochondria isolation

Mitochondrial and cytosolic fractions from IAV-infected or uninfected WT BMD-Mφ were extracted using Qproteome Mitochondria Isolation Kit (Qiagen) following manufacturer’s intructions. Purified mitochondria were lysed with RIPA buffer and further analysed by western blot using antibodies against RIPK3, CYPD, MAVS and actin.

### Immunoprecipitation

Protein (250–500μg) from whole-cell extracts lysed in lysis buffer (1% Triton X-100, 150mM NaCl, 20mM Hepes pH7.5, 10% glycerol, 1mM EDTA, supplemented with anti-protease cocktail, Roche) were incubated with anti-RIPK3 or anti-RIPK1 or rabbit IgG overnight at 4°C with gentle agitation. Protein G beads were then added for an additional 2 hours incubation at room temperature. Immunoprecipitates were washed 3 times with lysis buffer (2500g, 3min, 4°C), eluted by boiling in 2x Laemmli buffer and analyzed by western blot using antibodies against MAVS, RIPK3 or RIPK1.

### Confocal microscopy

BMD-Mφ were seeded in a media chamber of a glass microscopy slide (Nunc Labtek II). Cells were infected with IAV-PR8 for the indicated period of time and then fixed in 4% (vol:vol) paraformaldehyde for 15 min. When indicated, mitochondria were stained using Mitotracker Orange CMTMRos (200nM, Life Technologies #M7510) for 30 minutes at 37°C and then fixed with PFA. Cells were then permeabilized by incubating with 0.1% Triton X-100 in PBS for 15 min. Samples were blocked with 1% milk in PBS Tween 0.1% for 1h and then incubated with a specific rabbit polyclonal anti-IFN-β (1:50, #PA5-20390, ThermoFisher), anti-RIPK3 (1:50), anti-RIPK1 (1:50) or anti-pPKR (1:50) overnight at 4°C. Cells were incubated for 1 hour with secondary antibody Alexa Fluor 488- or Alexa Fluor 555-conjugated goat anti-rabbit (1:1000, Invitrogen) and nuclei were stained with Hoechst (1:2000, Molecular Probes). Coverslips were mounted (ProLong Gold Anti Fade, Invitrogen) onto microscope slides. Images were acquired using a Zeiss LSM 700 laser-scanning confocal microscope.

### Adoptive transfer model of infection

BMD-Mφ from Wild Type and *Ripk3*^-/-^ mice were generated as described previously. On day 7 of differentiation, BMD-Mφ were harvested and resuspended at a density of 1 x 10^6^ cells per 50μL. BMD-Mφ were then transferred by the intratracheal route into naïve *Rag1*^*-/-*^ mice. After 2 hours, *Rag1*^*-/-*^ were intranasally infected with 500 PFU of IAV. Lungs were harvested and processed as previously described for viral load analysis.

### Interferon-β treatment

Recombinant murine interferon-β was purchased from R&D Systems (#8234-MB-010). Mice were intranasally infected with 50 pfu of IAV. On day 2 post-infection, mice were given intranasally either PBS or IFN-β (2000U). Mice were euthanized on day 3 post-infection and lungs were harvested and processed to determine pulmonary viral load as previously described.

### Statistical analysis

Data are presented as mean ± SEM. Statistical analyses were performed using GraphPad Prism v6.0g software (Graphpad). Statistical differences were determined using log-rank test (survival studies: Fig 1B and S1B Fig), Kruskal-Wallis followed by Dunn’s multiple comparison test (Fig 4K), two-way ANOVA followed by Bonferroni’s multiple comparison test, or Mann-Whitney test, with significance expressed as follows: *p < 0.05, **p < 0.01, ***p < 0.001, ****p < 0.0001.

### Ethics statement

All experiments involving animals were approved by McGill (Permit # 2010–5860) in strict accordance with the guidelines set out by the Canadian Council on Animal Care. Human blood samples were collected from healthy donors following informed consent for the McGill University Health Centre (MUHC) institutional review board-approved research protocol GEN10-256.

## Supporting information

S1 Fig *Ripk3*^*-/-*^ mice are more susceptible to IAV. WT and *Ripk3*^*-/-*^mice were infected with a lethal dose of IAV (90 pfu) and morbidity, as a percentage of original weight (A) and survival (B) were assessed. (C-E) Mice were infected with 50 pfu. (C) IFN-β levels in lung homogenates were measured by ELISA at 0 and 6 days post-infection. (D) Percentage of NP^+^ pulmonary Mφ (CD45.2^+^ F4/80^+^ CD19^-^ cells) in the lungs of IAV-infected mice at 3 days post-infection. (E) Micrographs of H&E-stained lung sections 9 days after IAV infection. The inflammatory infiltrate (black arrows) was more prominent in *Ripk3*^*-/-*^ mice and mainly composed of intra-alveolar lymphocytes and histiocytes, with a few scattered neutrophils. Refers to Fig 1.
(TIFF)

S2 Fig Gating strategy to identify innate immune cells in the BAL of infected mice. Representative pseudocolour plots of the flow cytometry gating strategy of various innate leukocytes of the BAL of WT (A) and *Ripk3*^*-/-*^ (B) mice at 3 days post-infection with a sublethal dose of IAV. Total cell counts are quantified in Fig 1F. Numbers in proximity to each gate are the percentage of cells within that gate. Mφ are described as AM (Siglec F^+^, CD11c^+^, F4/80^+^) or IM (Siglec F^-^, CD11b^+^, GR1^-^, CD115^-^, F4/80^+^, CD11c^-^). Dendritic cells are considered Siglec F^-^, CD11b^+^, GR1^-^, CD115^-^, F4/80^-^, CD11c^+^ and neutrophils are Siglec F^-^, CD11b^+^, GR1^+^, CD115^-^. Monocytes are inflammatory (“Inflam Mono” Siglec F^-^, CD11b^+^, Gr1^+^, CD115^+^) or residential (“Res Mono” Siglec F^-^, CD11b^+^, Gr1^-^, CD115^+^).
(TIFF)

S3 Fig *Ripk3*^*-/-*^ Mφ are impaired in their anti-viral capacity, independent of cell death. (A) AMφ from WT and *Ripk3*^*-/-*^ mice were infected with IAV (MOI 5) and IFN-β levels were assessed in supernatants 24 hours post infection by ELISA. (B) BMDC from WT and *Ripk3*^*-/-*^ mice were infected with IAV (MOI 1) and IFN-β levels were assessed in supernatants by ELISA. (C) The viral load in culture supernatants of infected BMD-Mφ (MOI 1) was determined by standard plaque assay. (D) WT and RIPK3-deficient BMD-Mφ were pretreated with various combinations of zVAD (25μM) and Nec-1 (10μM) and LPS (100 ng/mL) for 24 hours. Cell death levels were assessed by LDH assay in cell culture supernatants. (E) The frequency of IAV-infected (MOI 1) Mφ undergoing apoptosis (Annexin V^+^, 7-AAD^-^) (top panel), or necrosis (Annexin V^-^, 7-AAD^+^) (bottom panel) was determined by flow cytometry, with representative zebra plots shown (left panels). The frequency of dead WT or *Ripk3*^*-/-*^ BMD-Mφ infected with IAV (MOI 1) was measured by flow cytometry, following staining with LIVE/DEAD dye (F). (G) WT and *Ripk3*^*-/-*^ mice were infected with IAV (50 pfu) and the percentage of Mφ (F4/80^+^, CD19^-^) undergoing apoptosis (left panel) or necrosis (right panel) was determined in the BAL using the same assay as in E. Refers to Fig 2.
(TIFF)

S4 Fig RIPK3 regulates MAVS-dependent type IFN pathway activation. (A) WT and *Mavs*^*-/-*^ BMD-Mφ were infected with IAV at MOI of 5. Supernatants were collected for relative quantification of total active type I IFN (α and β) using B16-blue reporter cells. (B) Immunoprecipitations were performed with beads only or control IgG to ensure specificity. (C) Immunofluorescence analysis of colocalization of RIPK1 (green) and mitochondria (red) in uninfected WT and *Ripk3*^*-/-*^ BMD-Mφ. Nuclei were stained with Hoechst (blue). Scale bar = 10μm in relation to Fig 3C–3E. (D-F) WT and *Ripk3*^*-/-*^ BMD-Mφ were transfected, or not, with 1μg/mL of the RIG-I ligand 5’ppp dsRNA. (D) Following transfection, interaction of RIPK1 with MAVS was determined as in Fig 3E. (E) Phosphorylation of IRF3 was determined by western blot (n = 3) and densitometry analysis of the ratio of pIRF3 on total IRF3 is shown on right panel. (F) Expression of antiviral IFN-β mRNA was assessed by qPCR. (G) Densitometry analysis of the ratio of pTBK1 on total TBK1 related to Fig 3H.
(TIFF)

S5 Fig RIPK3 activates PKR in IAV-infected Mφ to stabilize IFN-β mRNA. (A) Densitometry analysis of the expression of PKR at different time points in WT and *Ripk3*^*-/-*^ BMD-Mφ infected with IAV (MOI 5). (B-C) Difference in the expression of *GAPDH* (B) and *IL-6* (C) between WT and *Ripk3*^*-/-*^ BMD-Mφ infected with IAV. Gene expression was analyzed by qPCR following cDNA generation using random hexamers (blue bars) or oligo(dT) primers (white bars), as in Fig 4F.
(TIFF)
