# Supplementary figures and images for: RIPK3 interacts with MAVS to regulate type I IFN-mediated immunity to Influenza A virus infection
Source: PLoS Pathog. 2017 Apr 14;13(4):e1006326. doi: 10.1371/journal.ppat.1006326 (PMC5406035; doi:10.1371/journal.ppat.1006326)

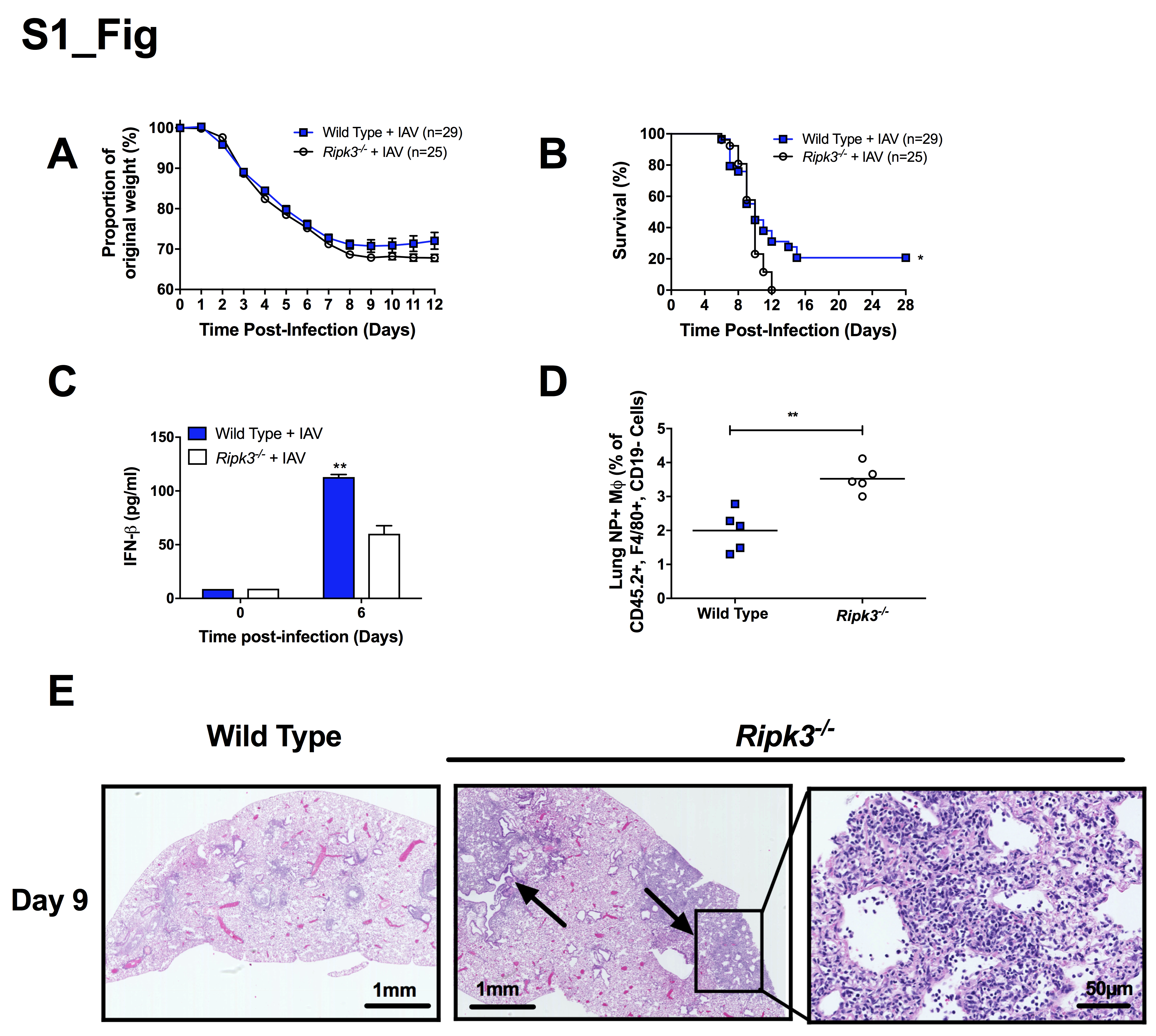

Supplement: S1 Fig — WT and Ripk3-/-mice were infected with a lethal dose of IAV (90 pfu) and morbidity, as a percentage of original weight (A) and survival (B) were assessed. (C-E) Mice were infected with 50 pfu. (C) IFN-β levels in lung homogenates were measured by ELISA at 0 and 6 days post-infection. (D) Percentage of NP+ pulmonary Mφ (CD45.2+ F4/80+ CD19- cells) in the lungs of IAV-infected mice at 3 days post-infection. (E) Micrographs of H&E-stained lung sections 9 days after IAV infection. The inflammatory infiltrate (black arrows) was more prominent in Ripk3-/- mice and mainly composed of intra-alveolar lymphocytes and histiocytes, with a few scattered neutrophils. Refers to Fig 1. (TIFF) [file ppat.1006326.s001.tiff]

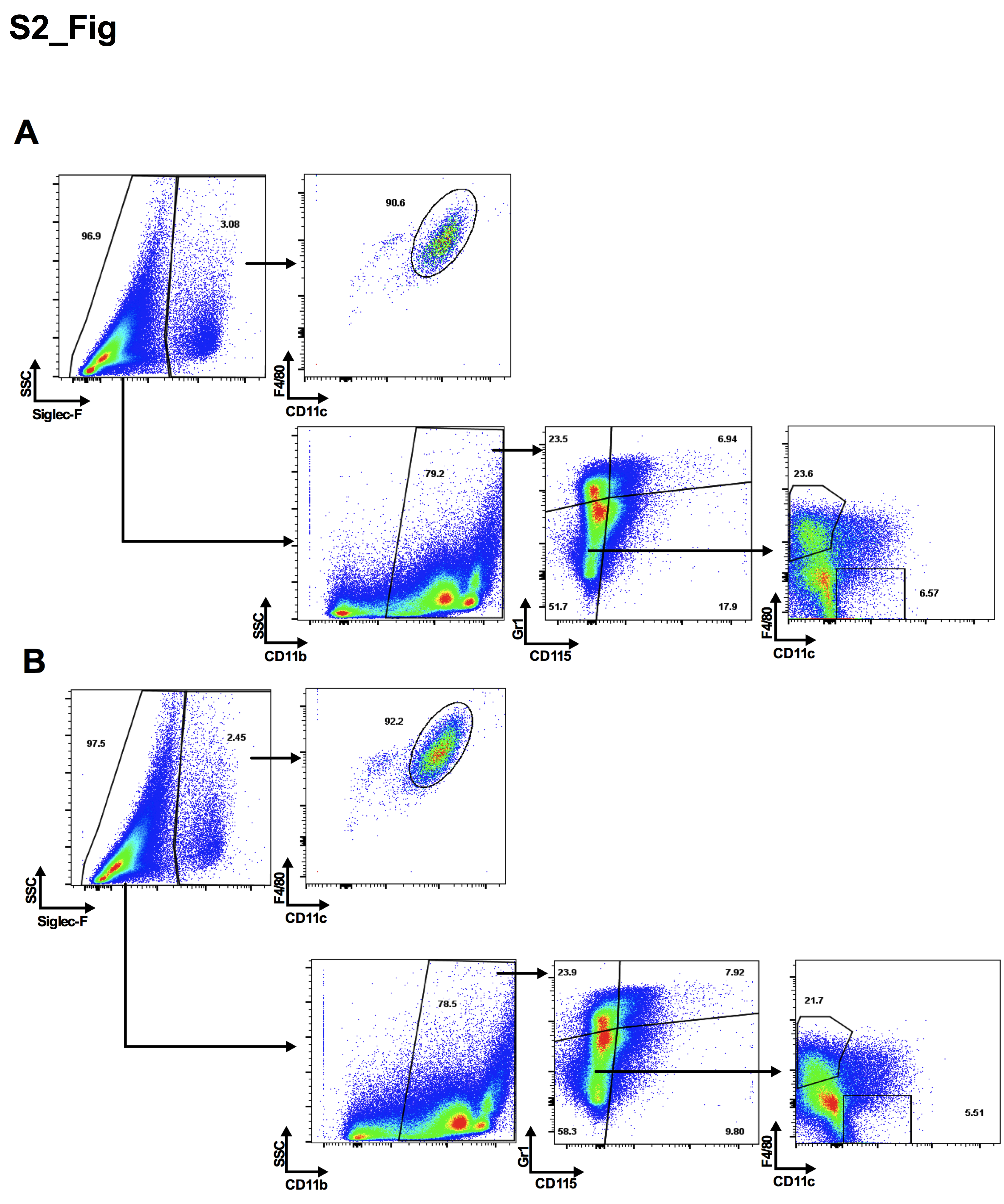

Supplement: S2 Fig — Representative pseudocolour plots of the flow cytometry gating strategy of various innate leukocytes of the BAL of WT (A) and Ripk3-/- (B) mice at 3 days post-infection with a sublethal dose of IAV. Total cell counts are quantified in Fig 1F. Numbers in proximity to each gate are the percentage of cells within that gate. Mφ are described as AM (Siglec F+, CD11c+, F4/80+) or IM (Siglec F-, CD11b+, GR1-, CD115-, F4/80+, CD11c-). Dendritic cells are considered Siglec F-, CD11b+, GR1-, CD115-, F4/80-, CD11c+ and neutrophils are Siglec F-, CD11b+, GR1+, CD115-. Monocytes are inflammatory (“Inflam Mono” Siglec F-, CD11b+, Gr1+, CD115+) or residential (“Res Mono” Siglec F-, CD11b+, Gr1-, CD115+). (TIFF) [file ppat.1006326.s002.tiff]

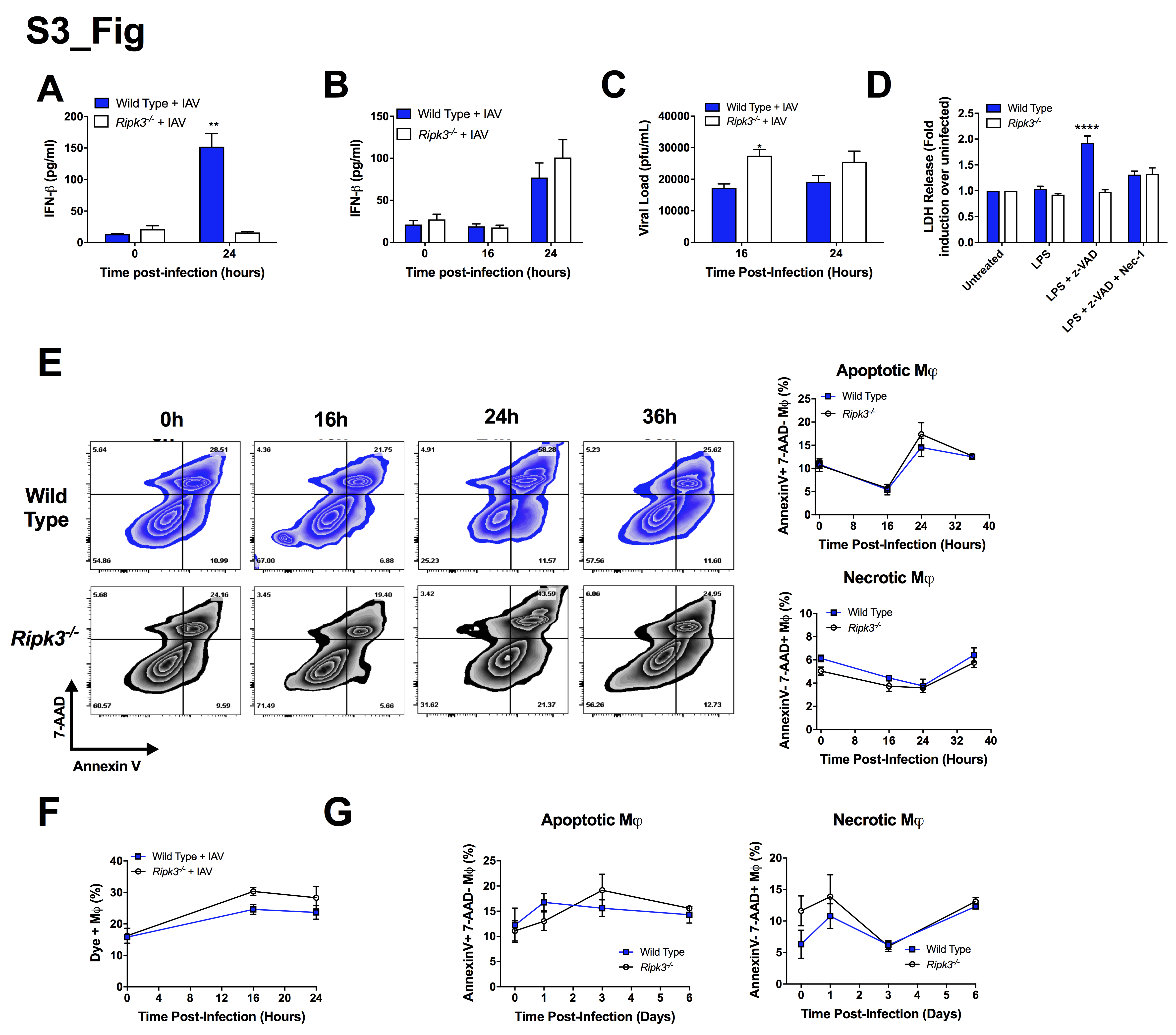

Supplement: S3 Fig — (A) AMφ from WT and Ripk3-/- mice were infected with IAV (MOI 5) and IFN-β levels were assessed in supernatants 24 hours post infection by ELISA. (B) BMDC from WT and Ripk3-/- mice were infected with IAV (MOI 1) and IFN-β levels were assessed in supernatants by ELISA. (C) The viral load in culture supernatants of infected BMD-Mφ (MOI 1) was determined by standard plaque assay. (D) WT and RIPK3-deficient BMD-Mφ were pretreated with various combinations of zVAD (25μM) and Nec-1 (10μM) and LPS (100 ng/mL) for 24 hours. Cell death levels were assessed by LDH assay in cell culture supernatants. (E) The frequency of IAV-infected (MOI 1) Mφ undergoing apoptosis (Annexin V+, 7-AAD-) (top panel), or necrosis (Annexin V-, 7-AAD+) (bottom panel) was determined by flow cytometry, with representative zebra plots shown (left panels). The frequency of dead WT or Ripk3-/- BMD-Mφ infected with IAV (MOI 1) was measured by flow cytometry, following staining with LIVE/DEAD dye (F). (G) WT and Ripk3-/- mice were infected with IAV (50 pfu) and the percentage of Mφ (F4/80+, CD19-) undergoing apoptosis (left panel) or necrosis (right panel) was determined in the BAL using the same assay as in E. Refers to Fig 2. (TIFF) [file ppat.1006326.s003.tiff]

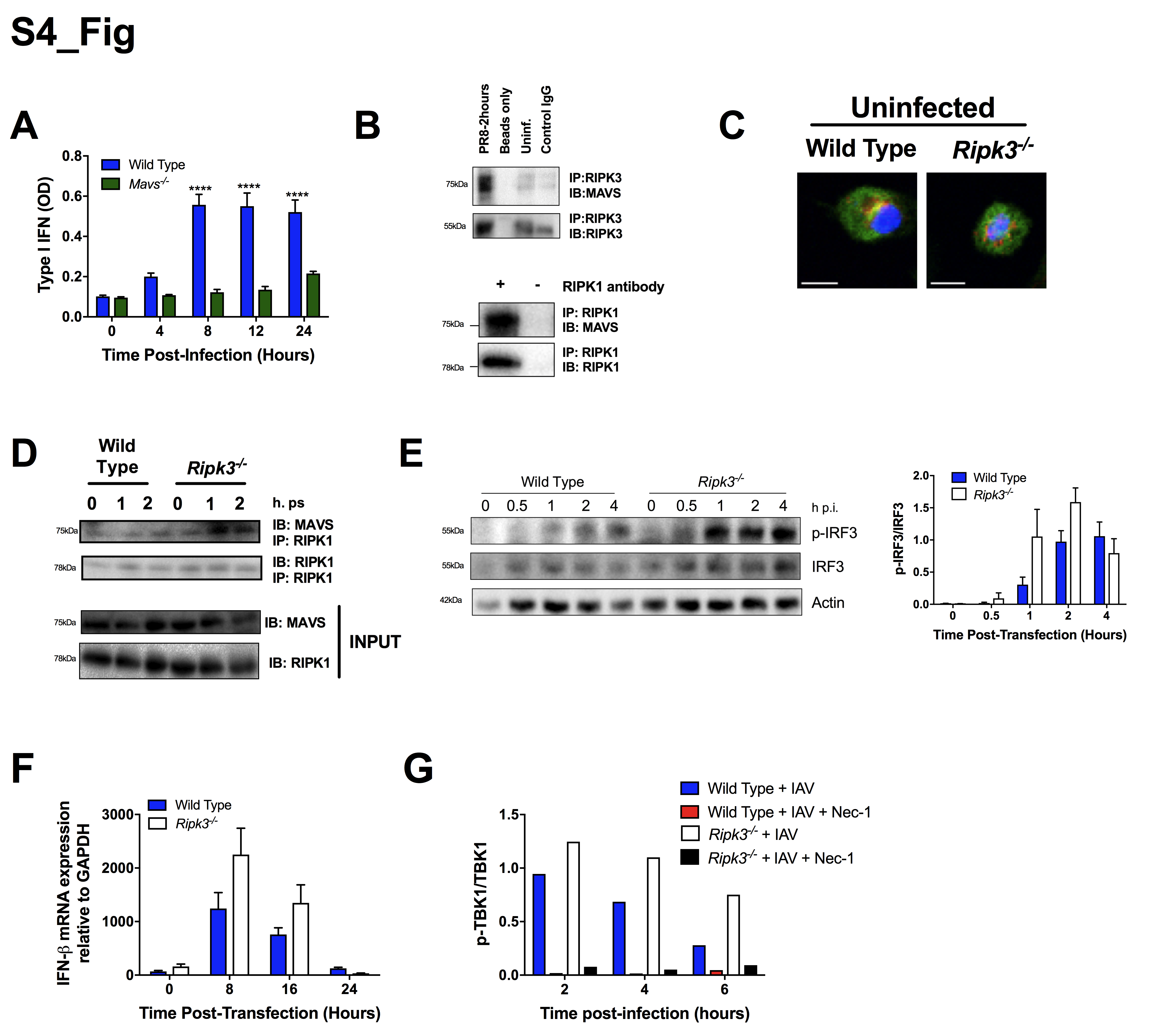

Supplement: S4 Fig — (A) WT and Mavs-/- BMD-Mφ were infected with IAV at MOI of 5. Supernatants were collected for relative quantification of total active type I IFN (α and β) using B16-blue reporter cells. (B) Immunoprecipitations were performed with beads only or control IgG to ensure specificity. (C) Immunofluorescence analysis of colocalization of RIPK1 (green) and mitochondria (red) in uninfected WT and Ripk3-/- BMD-Mφ. Nuclei were stained with Hoechst (blue). Scale bar = 10μm in relation to Fig 3C–3E. (D-F) WT and Ripk3-/- BMD-Mφ were transfected, or not, with 1μg/mL of the RIG-I ligand 5’ppp dsRNA. (D) Following transfection, interaction of RIPK1 with MAVS was determined as in Fig 3E. (E) Phosphorylation of IRF3 was determined by western blot (n = 3) and densitometry analysis of the ratio of pIRF3 on total IRF3 is shown on right panel. (F) Expression of antiviral IFN-β mRNA was assessed by qPCR. (G) Densitometry analysis of the ratio of pTBK1 on total TBK1 related to Fig 3H. (TIFF) [file ppat.1006326.s004.tiff]

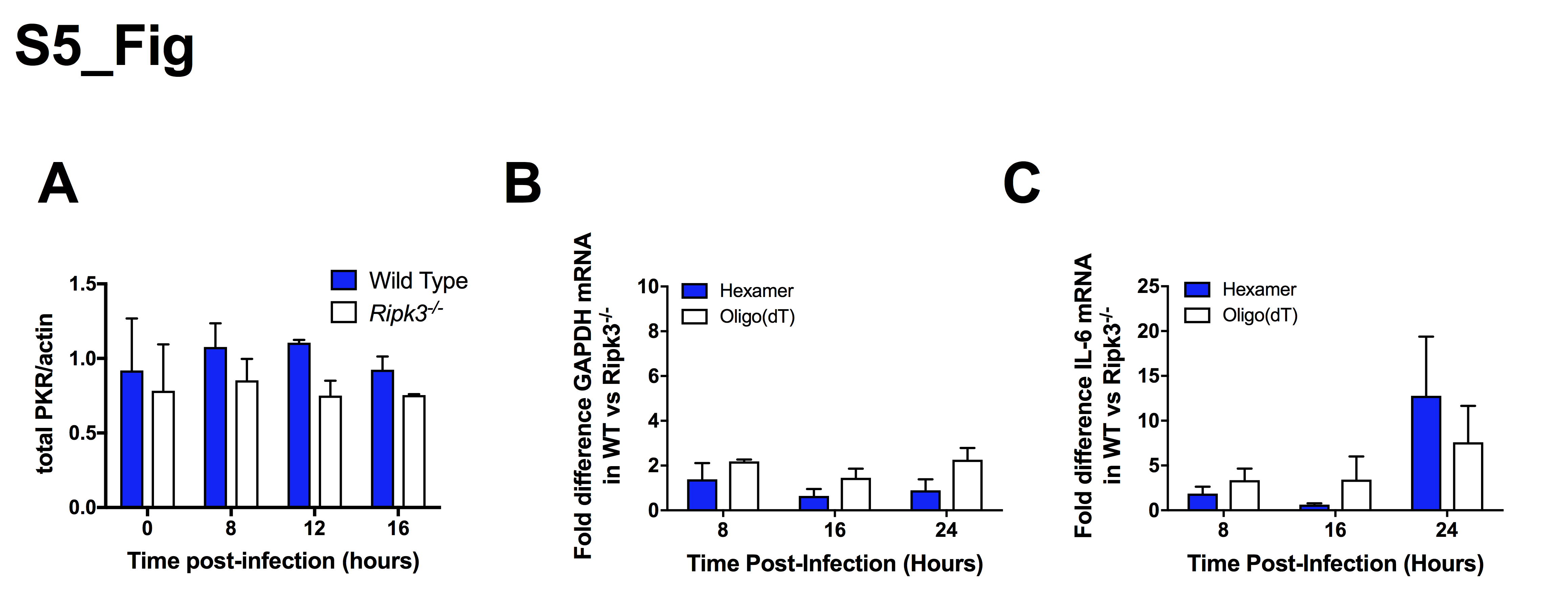

Supplement: S5 Fig — (A) Densitometry analysis of the expression of PKR at different time points in WT and Ripk3-/- BMD-Mφ infected with IAV (MOI 5). (B-C) Difference in the expression of GAPDH (B) and IL-6 (C) between WT and Ripk3-/- BMD-Mφ infected with IAV. Gene expression was analyzed by qPCR following cDNA generation using random hexamers (blue bars) or oligo(dT) primers (white bars), as in Fig 4F. (TIFF) [file ppat.1006326.s005.tiff]
